# Wood outlasts graphite: revolutionary birch biomass-based carbon anodes for long-life lithium-ion batteries

**DOI:** 10.1039/d6ra03501a

**Published:** 2026-06-04

**Authors:** Glaydson Simoes dos Reis, Mukhtiar Ahmed, Luis O. P. Silva, Jyri-Pekka Mikkola, Lashari Najeeb ur Rehman

**Affiliations:** a Laboratory of Industrial Chemistry and Reaction Engineering, Faculty of Science and Engineering, Åbo Akademi University 20500 Åbo/Turku Finland Glaydson.SimoesdosReis@abo.fi najeeb.lashari@abo.fi; b Wallenberg Wood Science Center, Technical Chemistry, Department of Chemistry, Chemical-Biological Centre, Umeå University SE-90187 Umeå Sweden; c Universidad de La Costa, CUC Calle 58 # 55–66 Barranquilla Atlántico Colombia

## Abstract

Here, we present carbon anode materials derived from birch biomass (BCAM) with hierarchical micro–mesoporous structure, largely amorphous carbon framework and abundant active sites for lithium storage and efficient ion-transport – prepared through phosphoric acid activation followed by high-temperature pyrolysis at 1000 °C. BCAM materials deliver a reversible capacity of 915 mA h g^−1^ at 1C after 1000 cycles, maintain 412 mA h g^−1^ after 2000 cycles at 2.5C, and retain 235 mA h g^−1^ after 5000 cycles at 5C with *ca*. 100% coulombic efficiency. As for comparison, a cell made with commercial graphite as also tested, the results showed a reversible capacity of 365 mA h g^−1^ at 1C after 400 cycles, a much lower capacity than that of BCAM anode material. Kinetic analysis indicates a substantial pseudocapacitive contribution, particularly at higher scan rates, which supports fast charge–discharge behavior. In addition, electrochemical impedance spectroscopy reveals low charge-transfer resistance and favorable lithium-ion diffusion kinetics. In total, these birch-derived carbons provide a steppingstone in robust, low-cost, and environmentally benign anode materials, and offer a promising route towards long-life time and sustainable lithium-ion batteries (LIBs).

## Abstract 2

Birch derived carbon anode materials (BCAM) with show hierarchical micro–mesoporous structure, mostly amorphous carbon framework offering abundant active sites for lithium storage and efficient ion-transport.

## Graphical abstract

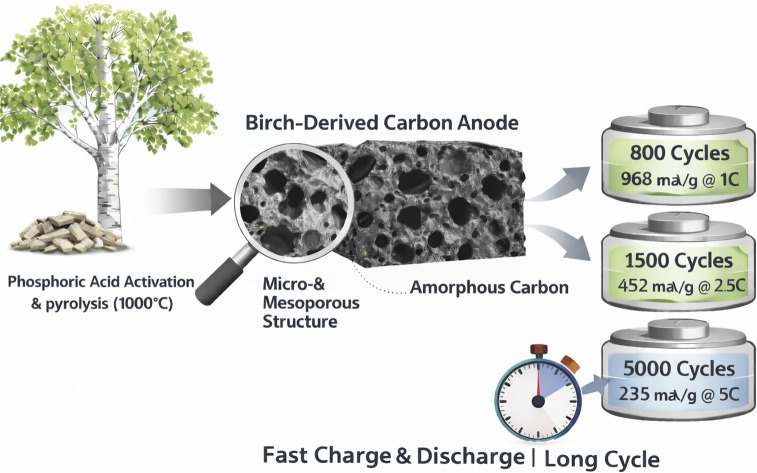

## 1. Introduction

The preparation of advanced sustainable materials from abundant biomass resources such as LIBs electrodes is a hot topic of current research – aligns the development of more sustainable energy storage systems with the advanced principles of circular economy.^1,2^ This evolution in sustainable LIBs explores the great potential of a wide range of organic materials from different origins such as plant, animal and industrial waste to create innovative materials for electrochemical application.^3,4^ The vital motivation to use these sources lies in the incessant search for greener alternatives to reduce or completely eliminate the structural dependence on non-renewable materials, such as mineral graphite, lithium and cobalt, the mining of which leads to vast and significant environmental impacts, elevated consumption of energy and, besides, are prone to great challenges in terms of geopolitical supply chains due their vast reserves being dominated by only few countries.^5^

One such possibility is to transform biomass-based resources (from agricultural, forestry and urban waste) into effective anode materials, whereupon this approach provides objectives to give these biomass resources a new, high-value life cycle, contributing to waste reduction and creation of a greener and potentially more accessible battery value chain.^6^ From biomass, carbon materials can be obtained – often through a controlled pyrolysis process.^7^ Carbon materials can be produced and tailored to have specific physical characteristics, which include well-developed high surface area and a combination of pore size structures that are vital for anode performance, where they are crucial as the Li^+^ ions hosts.^8^ However, there are some challenges to be faced regarding the use of biomass to tailor them as viable LIBs anodes, and are mainly related to improved electrochemical performance (capacity, cyclability, and charge rate), and techno-economic feasibility of scaling up the anode production from a different types of biomasses – ensuring consistent reproducibility and quality for industrial application is largely needed.

In brief, the production of battery components from biomass represents a sustainable and greener strategic line of research that aligns greater diversification of battery material sources and a reduction in CO_2_ footprint. Herein, we propose an easy and sustainable synthesis method of birch biomass carbon anode material preparation for LIBs. BCAM materials were prepared by using phosphoric acid activation method and high-temperature (1000 °C) pyrolysis and anode material was evaluated with respect to their electrochemical performance, such as cycling stability and rate capability.

## 2. Material and methods

### 2.1. BCAM preparation

**Fig. 1:**
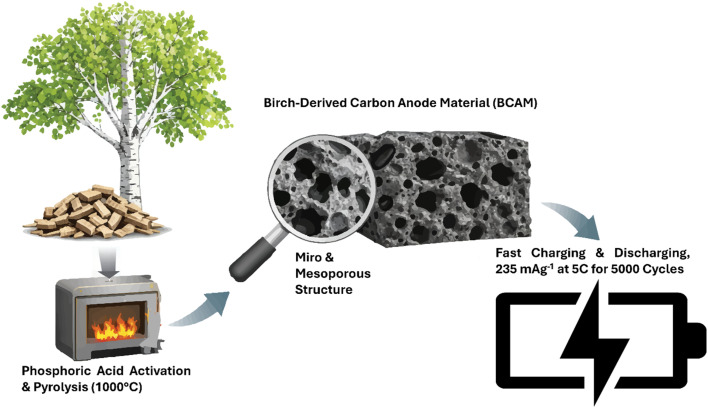
Schematic illustration of the synthesis and electrochemical performance of the birch-derived carbon anode material (BCAM).

BCAM prepared following the synthesis route schematically illustrated in Fig. 1 (disclaimer: some parts of figure are drawn by trained generative artificial intelligence). In a typical procedure, birch biomass was first immersed in a 2 M phosphoric acid (H_3_PO_4_) solution and transferred to a hydrothermal reactor, where the activation process was carried out at 150 °C for 12 h, which promotes chemical activation and the development of a porous structure within the biomass. After completion of the hydrothermal treatment, the activated biomass was thoroughly dried to remove residual moisture. Subsequently, the dried precursor was subjected to high-temperature pyrolysis at 1000 °C for 12 h under a controlled nitrogen (N_2_) atmosphere. The inert environment prevents oxidation during carbonization and facilitates the formation of a stable, porous carbon framework, yielding BCAMs.

### 2.2. Material characterization

Nitrogen (N_2_) adsorption–desorption measurements were conducted at 77 K using a Micromeritics 3Flex Sorptometer to evaluate the textural properties of the BCAM. Prior to the measurements, the samples were degassed under vacuum at an elevated temperature for several hours to remove physically adsorbed moisture and gases. The specific surface area was calculated using the Brunauer–Emmett–Teller (BET) method in the relative pressure (*P*/*P*_0_) range of 0.05–0.30. The total pore volume was estimated from the amount of N_2_ adsorbed at a relative pressure close to unity (*P*/*P*_0_ ≈ 0.99), while the pore size distribution was determined using the Barrett–Joyner–Halenda (BJH) method from the adsorption branch of the isotherm. The surface morphology and microstructural features of the BCAM were examined using scanning electron microscopy (SEM). Prior to imaging, the samples were mounted on conductive carbon tape. The crystalline structure and phase composition of the BCAM were analyzed by X-ray diffraction (XRD) using Panalytical Aeris. The diffraction patterns were recorded using Cu Kα radiation (*λ* = 1.5406 Å) over a 2*θ* range of typically 10–80°, with an appropriate scanning rate. The XRD patterns obtained were used to identify crystalline phases and to assess the degree of crystallinity of the material. Raman spectroscopy was employed to investigate the structural characteristics and bonding nature of the BCAM. Raman spectra were collected using a laser excitation source (commonly 532 nm or 633 nm) within a spectral range of 200–3000 cm^−1^. The Raman analysis provided insights into the degree of graphitization, defect density, and disorder in the carbon structure, typically evaluated through the intensity ratio of the D and G bands. Thermogravimetric analysis (TGA) was conducted to evaluate the thermal stability using Discovery SDT 650 and compositional characteristics of the BCAM. The measurements were carried out by heating the sample from room temperature to a designated upper temperature (*e.g.*, 800–900 °C) at a constant heating rate (typically 10 °C min^−1^) under air. The TGA curves were analyzed to determine weight loss behavior, thermal decomposition characteristics, and residual content.

### 2.3. Electrode and cell preparation

The working electrodes were fabricated by mixing the BCAM active material, conductive acetylene black (NG04CO08030) as the conductive additive, and poly(vinylidene fluoride) (PVDF, NG08BE1101) as the binder (all purchased from Nanografi) in a weight ratio of 8 : 1 : 1 (wt : wt : wt). The obtained powder mixture was dispersed in *N*-methyl-2-pyrrolidone (NMP, Honeywell Fluka, Lot: S44046 34207B13, purity ≥99%) and magnetically stirred overnight to form a homogeneous slurry. The resulting slurry was uniformly coated onto copper foil current collectors (Canrd, China) using a doctor-blade technique with a blade thickness of 100 µm. After coating, the electrodes were dried in a vacuum oven at 80 °C overnight to completely remove the residual solvent. The dried electrode films were then punched into circular disks with a diameter of 15 mm and used as working electrodes.

CR2032-type coin cells were assembled in an argon-filled glovebox with oxygen and moisture levels maintained below 0.5 ppm. Lithium metal foil was employed as both the counter and reference electrode, while glass fiber served as the separator. The separator was fully soaked with 120 µL of electrolyte consisting of 1 M LiPF_6_ EC/EMC (purchased from Dodochem). The prepared BCAM electrodes were used as the working electrodes for all electrochemical measurements. For the graphite half-cell, commercial graphite (Fluka 78391) with particle sizes from 0 to 20 µm was used.

### 2.4. Electrochemical characterizations

Cyclic voltammetry (CV) and electrochemical impedance spectroscopy (EIS) measurements were conducted using a Bio-Logic electrochemical workstation. CV tests were performed within a potential window of 0.01–3.0 V *versus* Li/Li^+^ at scan rates ranging from 0.1 to 1.0 mV s^−1^. EIS measurements were carried out over a frequency range of 100 kHz to 0.01 Hz with an AC perturbation amplitude of 5 mV.

Galvanostatic charge–discharge (GCD) measurements were conducted using a LANHE battery testing system. The GCD tests were performed within the voltage window of 0.01–3.0 V *versus* Li/Li^+^, at different current densities, to evaluate the cycling performance and rate capability of the BCAM electrodes. Long-term cycling tests were conducted at selected current densities to assess the electrochemical stability of the electrodes over extended cycling. Specific capacities were calculated based on the mass of the active material in the working electrode.

GITT measurements were performed by applying a constant current pulse to the assembled half-cells for a fixed duration (*τ*), followed by an open-circuit relaxation period until the voltage approached a quasi-equilibrium state. The Li^+^ diffusion coefficient (*D*_Li^+^_) was calculated from the transient voltage response according to the classical GITT equation:
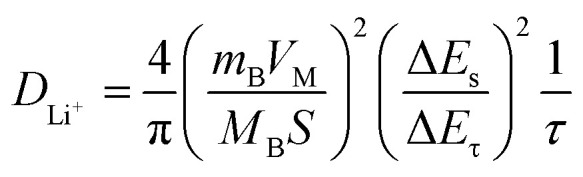
where *m*_B_ is the mass of active material, *M*_B_ is its molar mass, *V*_M_ is the molar volume, and S is the effective electrode surface area (approximated by the geometric area of the electrode). Δ*E*_τ_ represents the change in cell voltage during the current pulse after eliminating the initial ohmic drop, while Δ*E*_s_ is the steady-state voltage change between two successive relaxation steps. This method assumes semi-infinite diffusion conditions and small perturbations in lithium concentration during each pulse.

## 3. Results and discussion

### 3.1. Physicochemical properties of BCAM

**Fig. 2:**
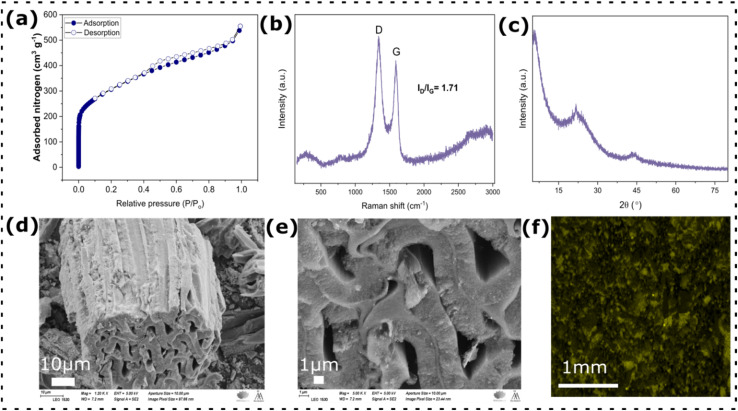
(a) N_2_ adsorption isotherm, (b) Raman spectrum showing G and D bands, (c) X-ray diffraction pattern of birch and (d and e) scanning electron microscopy images (f) shows the elemental mapping for phosphorus.

Birch biomass was converted into porous carbon through phosphoric acid activation and high-temperature pyrolysis at 1000 °C in a controlled environment. BCAMs exhibit a N_2_ adsorption isotherm (Fig. 2(a)), with two distinct patterns, first is associated to the presence of microporosity due to the high N_2_ adsorbed amounts, at low partial pressures (0.0 to 0.01 *P*/*P*_O_). The second is associated with the presence of hysteresis (between 0.4 and 0.9 *P*/*P*_O_), which indicates a presence of mesopores in its structure. Therefore, BCAMs exhibit a combined structure of micro-and mesoporous materials. The presence of both types of pores is highly beneficial because the micropores contributes to a high specific capacity and increase the cycling stability, while the mesopores contribute to a faster ion transport and a high-rate capability.^9–11^

Carbon's order/disorder level linked to its graphitization degree was evaluated by employing Raman spectroscopy (Fig. 2(b)), which shows the typical Raman for a biomass-derived carbons and evident signatures related to G and D bands centred at around 1345 and 1585 cm^−1^, respectively. These are related to the amorphous and/or disordered sp^2^-hybridized carbon defects, and to the crystalline lattice regarding graphitic structure, respectively.

For further evaluation, the *I*_D_/*I*_G′_ value was calculated. *I*_D_/*I*_G′_ can indicate whether the material possesses a more ordered graphitic structure or disordered carbon lattice.^12,13^ A higher *I*_D_/*I*_G_ value indicates that the carbon material has a more amorphous/defective carbon lattice, while a smaller *I*_D_/*I*_G_ value suggests a more graphitized material, and with *I*_D_/*I*_G′_ value of 1.71BCAM has an amorphous nature, which was complimented and conformed by XRD patterns (see Fig. 2(c)). Fig. 2(d and e) shows the scanning microscopy images of BCAM, whereas it displays denser structure with several holes/channels with what seems to be an extensive internal porosity yielding a significant SSA, in accordance with its high micro-meso porosity, which can ease the electrolyte/charge penetration in the internal anode structure. BCAM morphology seems to correlate with its porosity feature with developed pore network. Fig. 2(f) also displays element mapping of phosphorus element, which shows a homogeneous distribution over its structure, this could facilitate the contact between electrolyte/BCAM surface and improve Li^+^ storage performance. An amorphous material can have positive effect on LIBs performance.^14^ An amorphous one may facilitate the formation of a stable solid electrolyte interface (SEI) layers, which lead to an improved ion/electron transport.^15^ Moreover, generally, amorphous carbons have higher SSA, which increases the contact for Li-ion storage, and it helps to accommodate the high-volume expansion.^16^ Fig. S1 shows the thermogravimetric analysis (TGA) and differential thermogravimetric analysis (DTGA) curves of the BCAM anode. A small weight loss at low temperatures is observed, which can be attributed to the removal of physically adsorbed moisture and residual volatile species. The main weight-loss region at higher temperatures corresponds to the decomposition of organic components and the gradual oxidation of the carbon matrix. The DTGA peaks clearly indicate the temperatures at which the maximum mass-loss rates occur, confirming the thermal stability of the BCAM anode under typical electrode processing and operating conditions.

TEM was also employed to examine the carbon structure of BCAM (Fig. S2).

Fig. S2 shows typical high-resolution transmission electron microscopy (HRTEM) for carbon materials prepared at high temperature pyrolysis. HRTEM images show that a big amount of short and disordered graphite structure coexists with amorphous carbon, are consistent with Raman and XRD observations. Fig. S2(a) shows tangled fringes of carbon lattice with random directions what seems to be a pattern of hard carbon structure. This like-hard-carbon structure may facilitate the ions transport and boost the BCAM electrochemical performance.

**Fig. 3:**
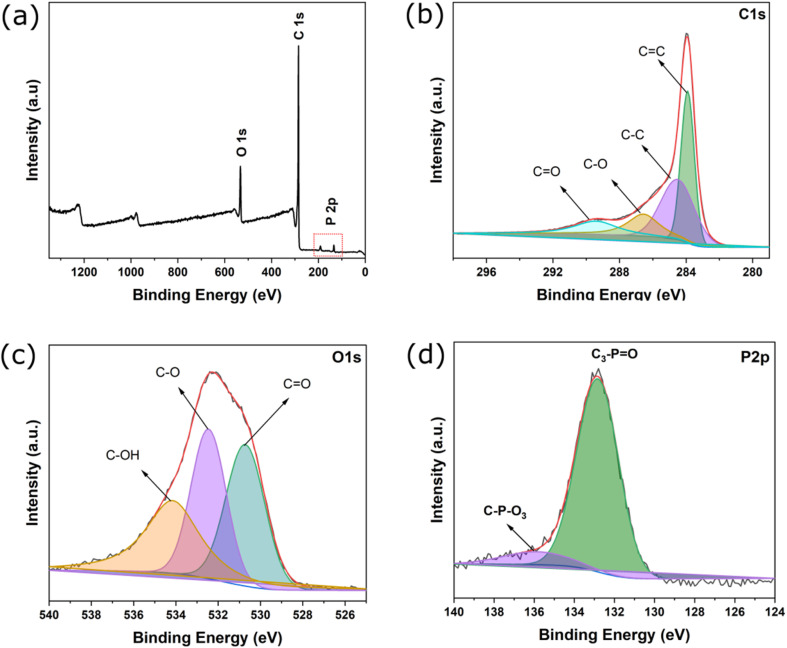
XPS analysis of BCAM: (a) full survey spectrum; (b) high-resolution C 1s spectrum; (c) high-resolution O 1s spectrum; and (d) high-resolution P 2p spectrum.

X-ray photoelectron spectroscopy (XPS) was employed to investigate the surface chemical composition of BCAM, as shown in Fig. 3. The survey spectrum (Fig. 3(a)) reveals distinct signals corresponding to C 1s, O 1s, and P 2p, confirming the successful incorporation of phosphorus and oxygen species into the carbon framework. The high-resolution C 1s spectrum (Fig. 3(b)) can be deconvoluted into four characteristic components, attributed to graphitic C=C, C–C bonds, and oxygen-containing functionalities such as C–O and C=O, indicating partial surface oxidation and heteroatom functionalization of the carbon matrix. In the O 1s spectrum (Fig. 3(c)), three main peaks centered at approximately 530.7, 532.4, and 534.2 eV are observed. These peaks correspond to C=O groups associated with carbonyl or quinone-type structures, C–O bonds in aromatic or ether configurations, and surface hydroxyl (C–OH) species, respectively, demonstrating the presence of abundant oxygen-containing functional groups. The high-resolution P 2p spectrum (Fig. 3(d)) further confirms phosphorus doping in BCAM, showing characteristic peaks assigned to C_3_–P=O and C–P–O bonding environments at around 132.9 and 135.7 eV, respectively.^17^ These phosphorus functionalities originate from the H_3_PO_4_ activation process and indicate the successful chemical integration of P atoms within the carbon matrix. The coexistence of oxygen- and phosphorus-containing surface groups is beneficial for improving electrolyte wettability and facilitating charge transfer, which can effectively enhance the electrochemical energy storage performance of BCAM.^18,19^

### 3.2. Electrochemical performance characterization

**Fig. 4:**
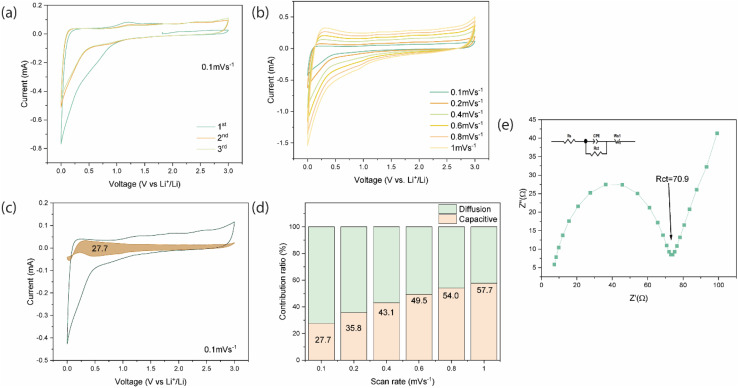
(a) Cyclic voltammetry (CV) curves of BCAM at a scan rate of 0.1 mV s^−1^ for the first three cycles, showing the activation behavior and stabilization upon cycling. (b) CV curves of BCAM recorded at different scan rates (0.1–1.0 mV s^−1^). (c) Separation of capacitive and diffusion-controlled charge contributions at 0.1 mV s^−1^, where the shaded region represents the capacitive contribution. (d) Histogram summarizing the capacitive contribution percentages of BCAM at various scan rates, showing an increase from 27.7% to 57.7% with increasing scan rate, confirming dominant pseudocapacitive behavior. (e) Nyquist plots (EIS) of BCAM.

The CV profile of BCAMs for the first three cycles, at a scan rate of 0.1 mV s^−1^ (Fig. 4(a)), showed almost overlapped CV profiles, underscoring its excellent electrochemical reversibility and structural stability in the first cycling process and highlights the absence of side-reactions between electrolyte and BCAM surface/structure, with only small redox peaks are observed during the first cycle, which are associated with the decomposition of the electrolyte and the formation of a solid-electrolyte interphase (SEI).

To further investigate the Li^+^ storage mechanism of BCAMs, CV curves at scan rates (from 0.1 to 1.0 mV s^−1^) were recorded (Fig. 4(b)) as the Li^+^ storage mechanism of a carbonaceous material is contributed by diffusion and capacitance,^20^ and the reaction kinetics can be studied by the change of CV curves at different scan rates. Here, the curves that the peak intensity increases with the increment of the scanning rate, with CV profiles showing minor shift of peak potential as well as wider anodic and cathodic peaks under higher scan rates and collectively do suggest a fast Li^+^ ions insertion/extraction.

To investigate the charge storage kinetics and distinguish the capacitive and diffusion-controlled contributions, cyclic voltammetry (CV) measurements were performed at different sweep rates. Using Dunn's method^21^ the relationship between the measured current response (*i*) and the scan rate (*v*) follows a power–law equation:*i* = *av*^*b*^where *a* and *b* are adjustable parameters. The *b*-value was determined from the slope of the plot of log(*i*) *versus* log(*v*). A *b*-value of 0.5 indicates a diffusion-controlled faradaic process associated with Li^+^ insertion, whereas a *b*-value of 1.0 corresponds to a surface-controlled capacitive process, including pseudocapacitive and electric double-layer contributions.

To quantitatively separate the capacitive and diffusion-controlled contributions, the current response at a fixed potential was expressed as:*i*(*V*) = *k*_1_*v* + *k*_2_*v*^1/2^where *k*_1_*v* corresponds to the capacitive contribution originating from surface-controlled processes, and *k*_2_*v*^1/2^ represents the diffusion-controlled Li^+^ insertion contribution. Rearranging the equation gives:
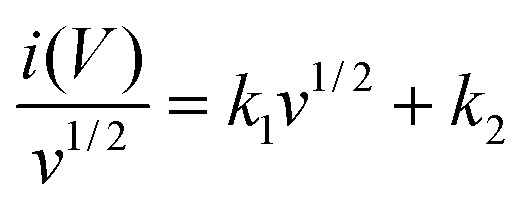


The constants *k*_1_ and *k*_2_ were determined from the slope and intercept of the linear plot of *i*(*V*)/*v*^1/2^*versus v*^1/2^ at each selected potential. This analysis enabled quantitative evaluation of the relative contributions from capacitive effects and diffusion-controlled Li^+^ insertion processes to the overall charge storage behavior. Fig. 4(c) shows the capacitive contribution, which is highlighted by the shaded region at 0.1 mV s^−1^ and indicates 27.7% of the Li^+^ storage on BCAM is a surface-controlled process even at low scan rates. The quantitative analysis of capacitive contribution of Li^+^ storage on BCAMs, at different scan rates (from 0.1 to 1 mVs^−1^) is shown in Fig. 4(d) and S3, which exhibits an increasing behavior from 27.7% at 0.1 mV s^−1^ to 57.7% at 1.0 mV s^−1^, confirming that a surface-controlled (pseudocapacitive) process is favored at higher scan rates, enabling a fast charge–discharge capacity.

Fig. 4(e) shows the Nyquist plot obtained from the EIS spectra of BCAMs. The semi-circle (high-frequency region) is associated with the charge transfer process at the electrode/electrolyte interface. A large semi-circle diameter means that the transfer resistance will also be large. BCAMs exhibited a small semicircle diameter, which indicates that BCAMs have a lower charge-transfer resistance (*R*_ct_), reflecting improved electrical conductivity and superior charge-transfer capability at the electrode–electrolyte interface, which can indicate high efficiency in storing Li^+^ ions. In the Nyquist plot, the oblique slope in the low-frequency region is attributed to the Warburg-type diffusion of Li^+^ into the electrode.^22^ The larger slope in the low-frequency region suggests a smaller barrier/impedance, leading to a higher diffusion coefficient of Li^+^ into the BCAM structure/surface. For a better comparison, the *R*_ct_ value of BCAM was 70.9 Ω, much smaller than 334 Ω, which is that of an activated carbon anode fabricated from a biomass of Calotropis gigantea.^23,24^ Another study produced a series of carbon materials to be employed as anodes for LIBs and their *R*_ct_ values varied from 128.8 to 217.5 Ω.

**Fig. 5:**
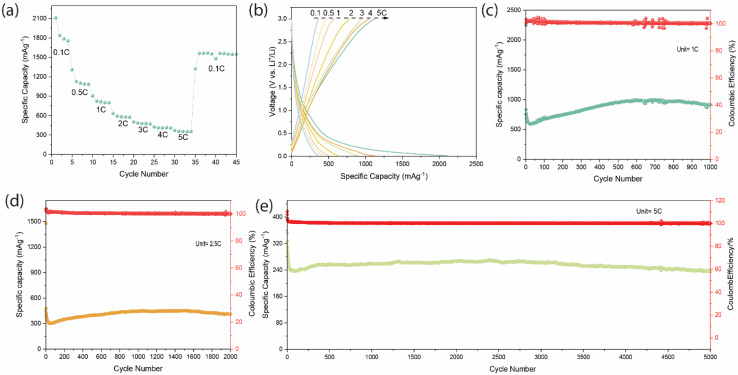
(a & b) rate performance and corresponding charge–discharge curves at 0.1, 0.5, 1, 2, 3, 4 and 5C respectively, (c) shows long cycle performance at 1C for 1000 cycles, (d) shows long cycle performance at 2.5C for 2000 cycles, (e) shows ultra-long cycle performance and Coloumbic efficiency at 5C for 5000 cycles.

Rate performance is an important parameter that measures on how effectively a battery delivers energy at different C-rates and further indicates its ability to support fast charging and high-power demands. Fig. 5(a) displays the rate capability of BCAMs at different C-rates. As expected, the capacity is decreased with increased C-rates (from 0.1C to 5C), and could be associated with the fact that redox reactions take place at higher extent on the surface of the BCAMs at faster charge–discharge rates (known as the C-rate effect), which hinder the participation of the total of active sites of BCAMs in storing Li^+^ ions. However, the capacity of the anode made an almost total recovery when it was subjected to the same initial 0.1C value. For the rate test, after 45 cycles, BCAMs returned to an extremely high-capacity of 1549 mA h g^−1^ at 0.1C, demonstrating their outstanding capacity reversibility. For comparison, rate performance of graphite anode is also shown in Fig. S5, which categorically demonstrates the outstanding capacity of BCAM material compared to graphite (see Fig. S5). The rate from low to high C-rate the graphite showed excellent recover (as well BCAM, see Fig. 5(c)), after 45 cycles delivered 410 mA h g^−1^, while BCAM delivered a recover capacity of 1564 mA h g^−1^, an impressive four times bigger of graphite at the end of rate capability test.

Fig. 5(a) shows the galvanostatic charge–discharge (GCD) profiles recorded at increasing C-rates (0.1–5 C) exhibit smooth, sloping voltage curves without distinct plateaus, which is characteristic of disordered carbon materials. The preservation of similar curve shapes with increasing current density, despite a gradual reduction in specific capacity and slightly increased polarization, indicates fast Li^+^ transport kinetics and good structural stability of the BCAM anode. This behavior is in good agreement with the rate capability displayed in Fig. 5(b), where the specific capacity decreases progressively as the C-rate increases from 0.1 to 5C due to kinetic limitations at higher currents. Importantly, when the current density is returned to 0.1C, the capacity almost fully recovers, demonstrating excellent reversibility and structural robustness. Such capacity recovery directly reflects the stable charge–discharge characteristics observed in Fig. 5(a), where no obvious distortion or degradation of the voltage profiles is detected even at high C-rates.

In comparison with other carbonaceous anode materials, such as graphite and conventional hard carbon, BCAMs exhibit a more stable voltage response and superior rate tolerance.^25^ While graphite typically shows sharp voltage plateaus and severe polarization at high rates, and many hard carbons suffer from sluggish Li^+^ diffusion,^26^ the combined results in Fig. 5(a) and 4(b) confirm that BCAMs maintain efficient Li^+^ insertion/extraction across a wide range of current densities. This enhanced performance can be attributed to the porous structure, abundant defect sites, and enlarged interlayer spacing of BCAMs, which collectively facilitate rapid ion transport and reversible Li^+^ storage.

The cycling stability of an anode material is of vital importance for the LIBs' performance. Fig. 5(c) illustrates the cycling performance of BCAMs at the end of 1000 cycles at 1C. The half-cell unfolded an exceptional performance of a discharge specific capacity of 915 mA h g^−1^ with a coulombic efficiency of 100%, which strongly indicate an outstanding cycling stability of the BCAMs. To better evaluate the effectiveness of BCAM anode, it was compared against the state-the-art anode material, a commercial graphite. The graphite cell delivered a discharge specific capacity of 365 mA h g^−1^ at 1C after 400 cycles (see Fig. S4), much lower than that of the BCAM capacity at this cycle number.

The results show extremely stable performances, BCAMs displayed 412 mA h g^−1^ after 2000 cycles at 2.5C (Fig. 5(d)). To further evaluate the cycle stability of BCAMs, a longer cycling performance was setup in Fig. 5(e) at higher current (5C) 235 mA h g^−1^ after 5000 cycles with CE close to 100%. These results suggest excellent long-cycle stability for BCAMs. A possible explanation of their excellent performance can be attributed to the combined micro-and meso pores structures, as well as their amorphousness that may have contributed to reduce the volume changes during the charge–discharge cycling.

Table 1 demonstrates that the BCAM delivers a significantly higher discharge capacity compared to previously reported biomass-derived anode materials. Based on this comparison, the present work exhibits the best overall lithium-ion battery performance among the listed studies. Notably, the BCAM is synthesized *via* a simple and straightforward route, rendering its preparation cost-effective and sustainable relative to state-of-the-art anode materials for LIBs. Furthermore, the results clearly show that the BCAM possesses excellent long-term cycling stability, highlighting its strong potential for practical application as a high-rate anode material in lithium-ion batteries.

**Table 1:** Comparison between BCAM anode with other works

Material	Rate capability	Cycling stability	Ref.
Lignin-derived porous carbon	420 mA h g^−1^ @ 0.2 A g^−1^	∼99% retention after 300 cycles; ∼85% after 1000 cycles	27
Rice husk-derived activated carbon	321 mA h g^−1^ @ 0.1 A g^−1^	∼81% retention; 400 cycles	28
Plane tree leaf porous carbon	243 mA h g^−1^ @ 2 A g^−1^	96–98% retention after ∼250 cycles (varied)	24
Nitrogen-rich porous carbon derived from ox horns	304 mA h g^−1^ @ 5 A g^−1^	90% retention beyond ∼100 cycles	29
Duck weed nitrogen doped porous carbon materials (SNCMs)	1071 mA h g^−1^ @ 100 mA g^−1^, and 630 mA h g^−1^ @ 1 A g^−1^	100% after 100 cycles, and 59% after 1000	30
Biomass to carbon nanofiber (CNF)	380 mA h g^−1^ @ 0.03 A g^−1^	55.89% after 200 cycles at 0.1 A g^−1^	31
Silicon-enriched biomass-derived carbon	83.8 mA h g^−1^ @ 1 g^−1^	80.8% after 2000 cycles	32
Birch biomass (BCAM)	915 mA h g^−1^ @ 1C, 412 mA h g @ 2.5C, and retain 235 mA h g^−1^ @ 5C	100% after 1000, 2000 and 5000 cycles	This work

**Fig. 6:**
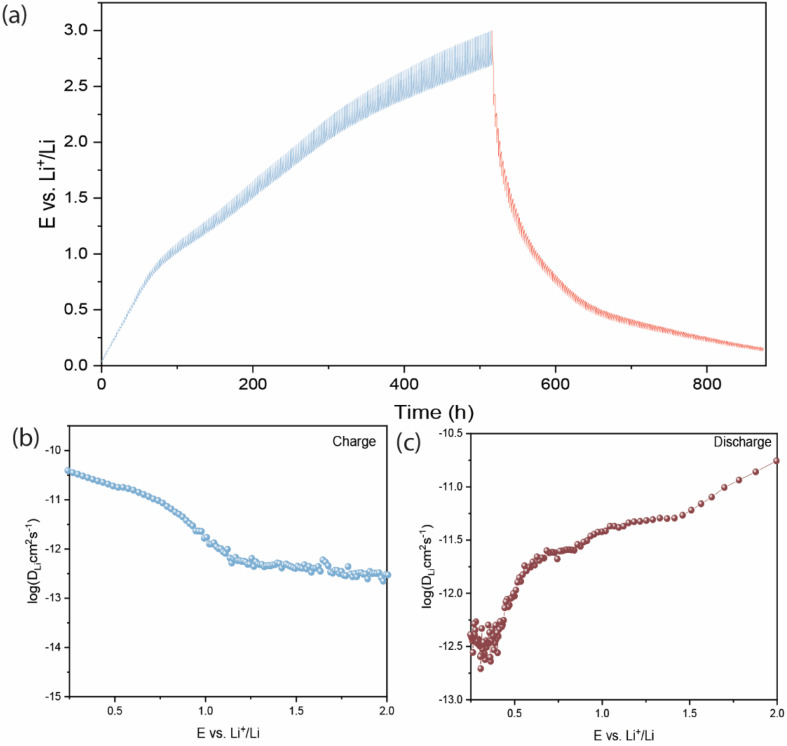
(a) GITT profile and calculated Li^+^ diffusion coefficient. Derived diffusion coefficients (*D*_Li^+^_), (b) charging, (c) discharging.

Fig. 6(a) shows the Time-dependent evolution of specific capacity and Li^+^ diffusion coefficient (*D*_Li_) of the electrode at 0.1C. In the region of 0.0–3.0 V, this plot illustrates the voltage response of the BCAM electrode as a function of time, highlighting the distinct plateaus that correspond to different stages of lithium-ion intercalation and deintercalation. The diffusion coefficients were determined from the GITT voltage transients, where Δ*E*_τ_ was extracted from the potential change during the current pulse after correcting for the ohmic drop, and Δ*E*_s_ was obtained from the steady-state voltage differences between successive relaxation steps for both charge and discharge processes, as illustrated in Fig. S7. The calculated log(*D*_Li_) across the potential ranges from 0.25 to 2.0 V range from 10^−10^ cm^2^ s^−1^ to 10^−13^ cm^2^ s^−1^ during charging (Fig. 6(b)) and discharging (Fig. 6(c)). BCAM electrodes shows an initial activation process with rapid stabilization of ion transport and diffusion coefficient, followed by a kinetically stable region and then a gradual degradation stage associated with reduced Li^+^ diffusivity.

The low Li^+^ diffusion coefficient (*D*_Li^+^_) indicates slow transport of Li ions within an electrode material, which directly causes high polarization, poor high-rate capability, and reduced capacity at high current densities. *D*_Li^+^_ is heavily influenced by the material's microstructure, specifically the specific surface area, which typically a higher surface area leads to a smaller diffusion coefficient.^33,34^ Thus, the low *D*_Li^+^_ presented by our BCAM, despite a high BET surface area, could be due to the increased surface interaction (adsorption), restricted pore geometry (tortuosity), and the presence of micropores.

## 4. Conclusions

In total, a high-performance and stable anode material was produced from birch tree biomass through the H_3_PO_4_ activation method. With high specific surface area material with combined micro-and-meso porosity features and amorphous nature. As the anode in LIBs, it shows the reversible Li^+^ intercalation, and delivered an extremely high specific capacity of 915 mA h g^−1^, at 1C after 1000 discharge–charge cycles, 412 mA h g^−1^ at 2.5C after 2000 discharge–charge cycles, and 235 mA h g^−1^ at 5.0C after 5000 discharge–charge cycles, with coulombic efficiency of *ca*. 100% at all C-rates, highlighting its very highly stable nature. When compared to commercial graphite (365 mA h g^−1^ at 1C after 400 cycles), BCAM exhibited a discharge capacity almost three times higher its value at the end of 100 cycles, which demonstrated its competitiveness front the dominant anode technology of LIBs nowadays, graphite. Such efficiency could be connected to their highly conductive network and the pore structure, and altogether BCAM can be considered a real promising anode material for LIBs due to their easy preparation and sustainable nature.

## Conflicts of interest

There are no conflicts to declare.

## Supplementary Material

RA-016-D6RA03501A-s001

## Data Availability

The data that support the findings of this study are available from the corresponding author upon reasonable request. Supplementary information (SI) is available. See DOI: https://doi.org/10.1039/d6ra03501a.
