# Supplementary material for: Wood outlasts graphite: revolutionary birch biomass-based carbon anodes for long-life lithium-ion batteries
Source: RSC Adv. 2026 Jun 4;16(33):30497–506. doi: 10.1039/d6ra03501a (PMC13237644; doi:10.1039/d6ra03501a)
Supplement: RA-016-D6RA03501A-s001 [file RA-016-D6RA03501A-s001.pdf]

Supplementary Information (SI) for RSC Advances.
This journal is © The Royal Society of Chemistry 2026

Supporting Information

Wood Outlasts Graphite: Revolutionary Birch-Based Bio-Carbon Anodes for Long-Life Lithium-Ion Batteries

Glaydson Simoes dos Reis[a], Mukhtiar Ahmed[b], Luis O.P. Silva[c], Jyri-Pekka Mikkola[a,b], Lashari Najeeb ur Rehman[a*]

[a-] *Laboratory of Industrial Chemistry and Reaction Engineering, Faculty of Science and Engineering, Åbo Akademi University, 20500, Åbo/Turku, Finland*

[b-] *Wallenberg Wood Science Center, Technical Chemistry, Department of Chemistry, Chemical-Biological Centre, Umeå University, SE-90187, Umeå, Sweden*

[c-] *Universidad de La Costa, CUC, Calle 58 # 55–66, Barranquilla, Atlántico, Colombia*

* Corresponding author

E-mail addresses: Glaydson.SimoesdosReis@abo.fi, najeeb.lashari@abo.fi

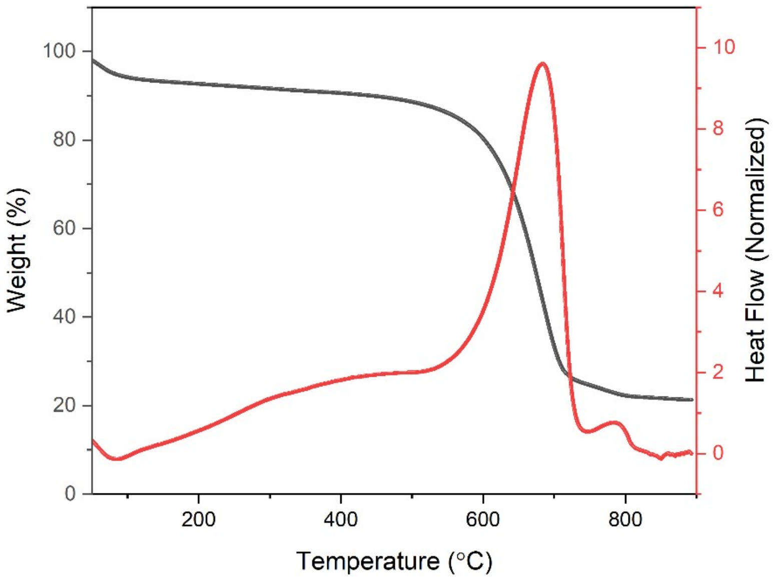

Figure S1 TGA and DTGA of BCAM

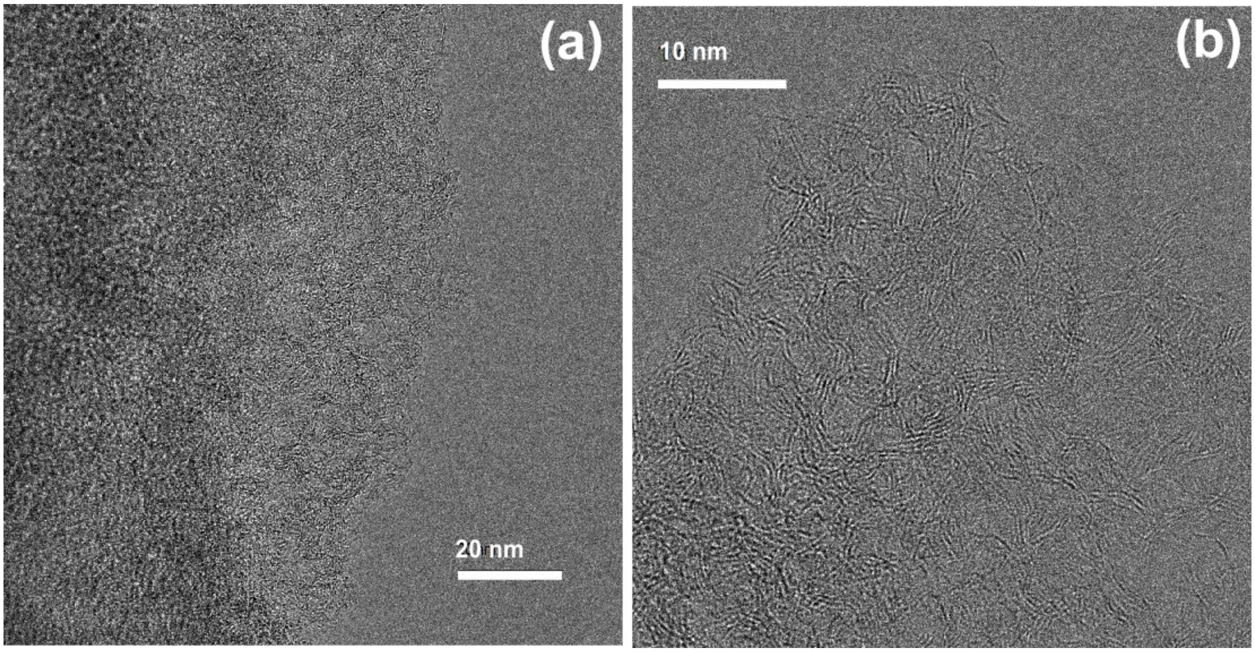

Figure S2 (a) TEM and (b) HRTEM of the BCAM

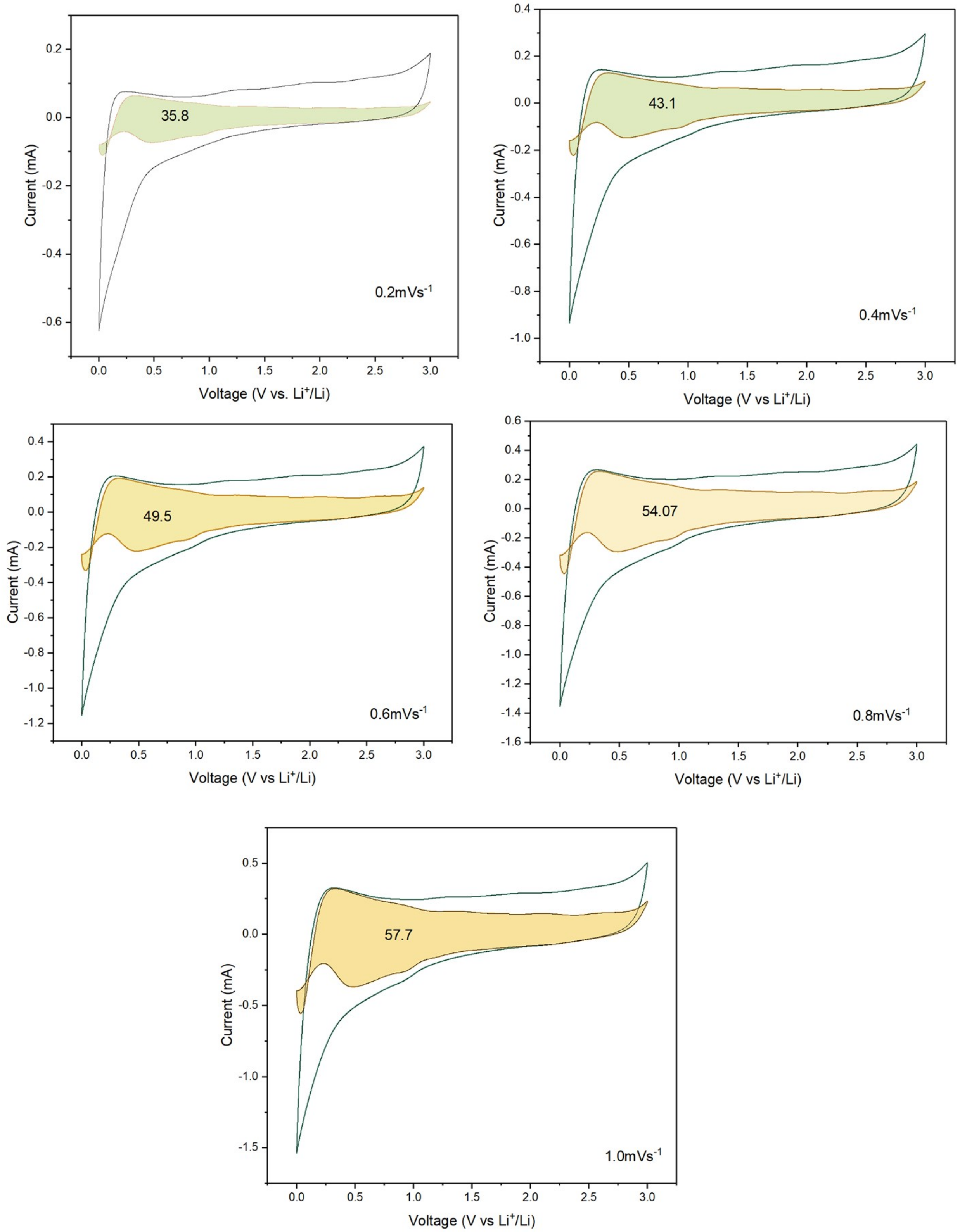

Figure S3 Capacitive and diffusion-controlled capacitance contribution of BCAMs at scan rates of 0.2, 0.4, 0.6, 0.8 and 1 mVs^{-1}

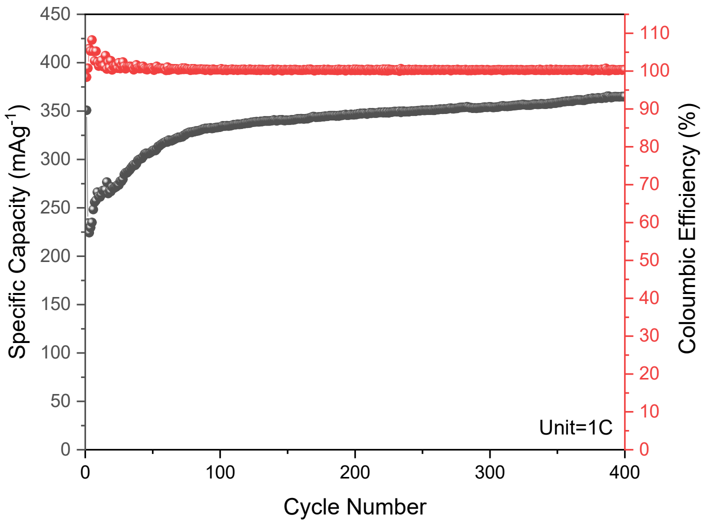

Figure S4. Long cycle performance of graphite at 1C for 400 cycles

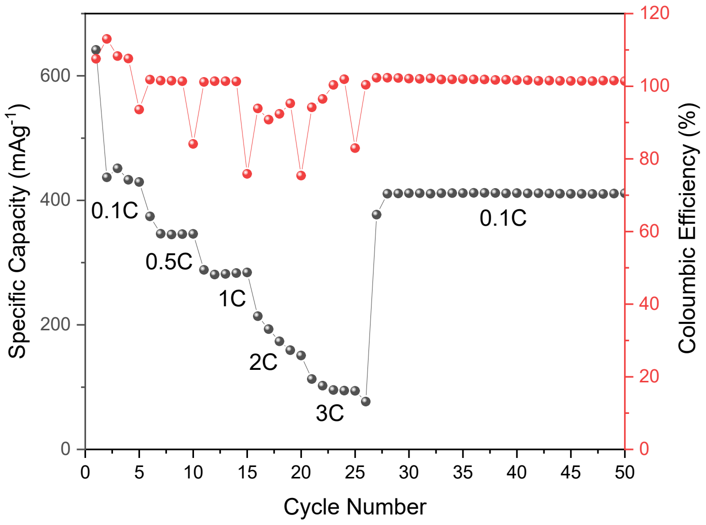

Figure S5 Shows the Rate performance of Graphite

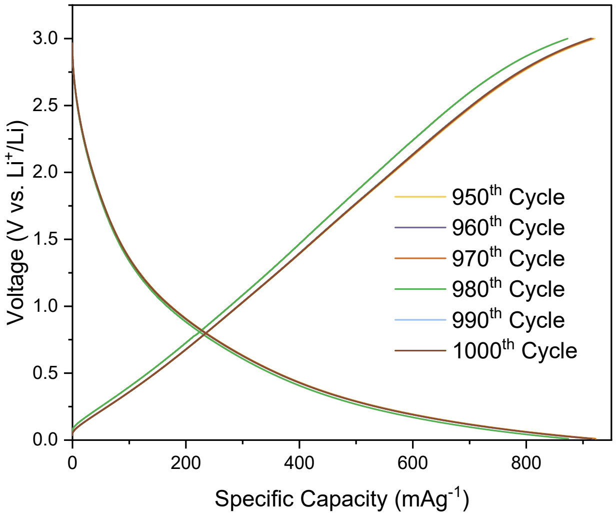

Figure S6 show charge-discharge curves for BCAM electrode at 1C.

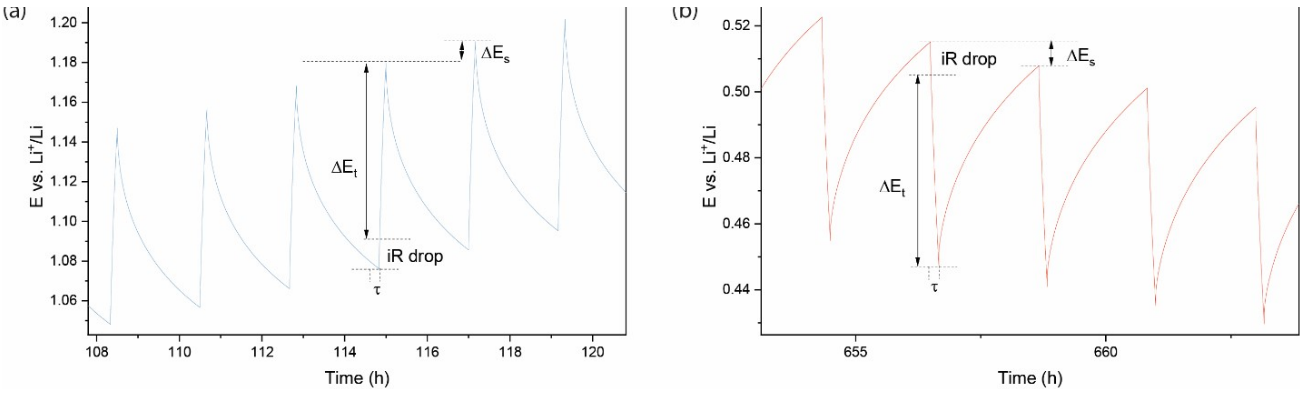

Figure S7. current step during (a)charge and (b)discharge in GITT curve
